# SARS-CoV-2 Viral and Serological Testing When College Campuses Reopen: Some Practical Considerations

**DOI:** 10.1017/dmp.2020.266

**Published:** 2020-07-27

**Authors:** Isaac Chun-Hai Fung, Chi-Ngai Cheung, Andreas Handel

**Affiliations:** Department of Biostatistics, Epidemiology, and Environmental Health Sciences, Jiann-Ping Hsu College of Public Health, Georgia Southern University, Statesboro, Georgia; Department of Psychology and Criminal Justice, Middle Georgia State University, Macon, Georgia; Department of Epidemiology and Biostatistics, College of Public Health, University of Georgia, Athens, Georgia

**Keywords:** communicable diseases, infectious disease transmission, social media, digital health, epidemics

## Abstract

The coronavirus disease 2019 (COVID-19) pandemic prompted universities across the United States to close campuses in Spring 2020. Universities are deliberating whether, when, and how they should resume in-person instruction in Fall 2020. In this essay, we discuss some practical considerations for the use of 2 potentially useful control strategies based on testing: (1) severe acute respiratory syndrome coronavirus 2 (SARS-CoV-2) reverse transcriptase-polymerase chain reaction (RT-PCR) testing followed by case-patient isolation and quarantine of close contacts, and (2) serological testing followed by an “immune shield” approach, that is, low social distancing requirements for seropositive persons. The isolation of case-patients and quarantine of close contacts may be especially challenging, and perhaps prohibitively difficult, on many university campuses. The “immune shield” strategy might be hobbled by a low positive predictive value of the tests used in populations with low seroprevalence. Both strategies carry logistical, ethical, and financial implications. The main nonpharmaceutical interventions will remain methods based on social distancing (eg, capping class size) and personal protective behaviors (eg, universal facemask wearing in public space) until vaccines become available, or unless the issues discussed herein can be resolved in such a way that using mass testing as main control strategies becomes viable.

In spring 2020, coronavirus disease 2019 (COVID-19) spread across the United States and impelled universities to switch to online instruction. Although the pandemic trajectory remains uncertain,^1^ scientific consensus is that the pandemic will continue into the fall.^2^ Many epidemiologists have warned of a rebound of cases, due in part to a relaxation of social distancing.^3,4^ One university president wrote recently, “The COVID-19 virus will remain a fact of life this autumn.”^5^ University student life, including classroom instruction, cafeterias, residential halls, and sport and cultural activities, provides ample opportunities for severe acute respiratory syndrome coronavirus 2 (SARS-CoV-2) transmission. Alleged “failure” to protect students from SARS-CoV-2 infection on campus carries legal risks too, as some may file class-action law suits against their universities, as in a recent example.^6^ To reopen campuses and avoid outbreaks this fall will require careful deliberation.

Anticipating the Fall Semester, universities are deliberating whether, and to what extent, they should reopen their campuses to in-person instruction.^7,8^ As of June 23, 64% of 1030 US colleges surveyed plan for in-person instruction, while 24% planned for online or hybrid models.^8^ As universities announce their intentions, all have placed public health considerations at the top of criteria for reopening. In May, the American College Health Association (ACHA) issued its guidelines on campus reopening,^9^ and the Centers for Disease Control and Prevention (CDC) published its “Considerations.”^10^ Both documents provide general information and infection control guidelines. Neither document discusses in detail the feasibility of using extensive viral or serological testing strategies, which have been proposed by some as potential important control strategies.^11,12^ To the best of our knowledge, the implementation of an extensive viral or serological testing program was unbeknownst to most university campuses before the COVID-19 pandemic. To further the debate on reopening university campuses for in-person instruction without a safe and effective COVID-19 vaccine, in this policy analysis, we discuss the practical aspects of implementing such testing strategies on college campuses (Table 1).

**TABLE 1:** Advantages and Disadvantages of Mass RT-PCR Testing for SARS-CoV-2 and Mass Serological Testing for Antibodies Against SARS-CoV-2

Testing	Pros	Cons
RT-PCR testing	Identify individuals currently infected with SARS-CoV-2 including asymptomatic individuals Provide information for monitoring the epidemic Public confidence When performed frequently enough, it might reduce transmission below the critical threshold of reproduction number = 1	Expensive Self-isolation and quarantining require large resources (buildings, personnel) Depending on the sensitivity or specificity of the test, potential issues with false positive and false negative results Legal issues with regard to restricting movement
Serological testing for antibodies	Identify individuals who have antibodies and who might be immune to reinfection Seroprevalence of the campus community Needs to be repeated less frequently than RT-PCR testing	Expensive Still unclear how test results (antibody levels) relate to protection against re-infection. Might lead to a false sense of safety. Poor test performance could lead to more transmission. Difficult ethical considerations regarding the “immune shield” option.

## RT-PCR Testing, Contact Tracing, Isolation, and Quarantine

Reverse transcriptase-polymerase chain reaction (RT-PCR) tests can be used to identify virus-shedding individuals. ACHA recommends campus-wide syndromic surveillance and that universities should ensure “access to immediate viral testing for all students, faculty, or staff with symptoms.”^9^ Given that SARS-CoV-2 can be transmitted presymptomatically,^13^ ACHA recommends “viral surveillance of asymptomatic students” where possible.^9^

As an example, the University of California San Diego is extending mass testing to asymptomatic individuals.^12^ The voluntary program will provide students with RT-PCR self-test kits and will test “60 to 90%” of the campus population “on a recurring basis.”^12^ Recurrent mass testing could be useful in detecting asymptomatic individuals and reducing transmission to others. For a basic reproduction number of 2.5,^14^ 2 of 3 potential transmissions must be prevented by prompt diagnosis and isolation to keep the reproduction number <1. Given an incubation period of around 5-6 days,^15,16^ and the assumption that the majority of transmission occurs after symptom onset,^13,17^ even testing everyone weekly, followed by rapid isolation and quarantine of infected individuals and their close contacts, respectively,^18^ might not be enough to prevent widespread campus transmission.

However, there are serious caveats. Mass testing is expensive.^19^ Medicare pays $100 for laboratory tests using high-throughput technologies for the detection of SARS-CoV-2.^20^ Using this pricing as a benchmark, each round of testing a campus of 10,000 individuals would cost 1 million dollars. Mass testing is logistically challenging, too. For example, to finish self-testing 10,000 individuals every week, a daily rate of self-testing 1429 individuals is needed. A staggered schedule of return and testing may make the logistics more manageable. Many universities may not have access to laboratories that can process a large number of specimens quickly enough so that results can be delivered in time to be relevant for contact tracing and isolation. Universities might not be able to rely on external laboratories for large testing programs because of cost and limits on throughput.

The program’s effectiveness in preventing SARS-CoV-2 transmission would depend upon high levels of voluntary participation, and a ready ability to support self-isolation and quarantine (eg, single residential rooms for isolated and quarantined students, staff monitoring of compliance, recording symptoms and clinical status, providing food and medical care). Many universities may lack the space and personnel needed to conduct such programs, especially during times when campus outbreaks are detected.^18^ Finally, many universities may lack the legal authority to enforce isolation and quarantine or otherwise to limit students’ freedom of movement.

Further challenges stem from the fact that tests are imperfect and produce both false negative and false positive results.^21,22^ False negative results may lead to otherwise avoidable transmission. Some rapid tests are reported to have a sensitivity as low as 85%,^23^ ie, there will be 15 false negatives for every 100 SARS-CoV-2 positive individuals. In programs relying on unsupervised self-collected nasal specimens, false negative results may arise from improper collection technique or transport. As long as SARS-CoV-2 prevalence on campus remains low, false positive results might be a larger concern, as the positive predictive value may be low unless the specificity is as high as 100%.^24^ This could lead to healthy students being misdiagnosed as being infected, and thus isolated, further exacerbating the resource and legal challenges mentioned above.

Specificity can be increased if 2 tests are required to confirm a case. However, this will substantially increase the cost and workload of mass testing. Specificity can also be increased if only symptomatic individuals are tested. However, because asymptomatic transmission is well documented,^13,25^ focusing on only symptomatic individuals is not an option if testing serves as the main control strategy. Overall, the profound logistical challenges of a test-and-isolate strategy prevent it from being the mainstay of outbreak control.

## Serological Testing for Antibodies and “Immune Shield” Strategy

Another testing strategy that has been proposed is to apply serological testing to identify individuals who have developed protective antibodies against SARS-CoV-2 and assign them as “immune shields.”^11^ Others have discussed the idea of issuing immunity certification,^26^ or immunity-based licenses,^27^ to individuals with antibodies against SARS-CoV-2. Assuming such individuals are immune to reinfection, they can be deployed to provide “shield immunity” to preferentially interact with susceptible or infected individuals, thus reducing transmission.^11,28^ A model showed that such an immune shielding approach could potentially lead to a reduction in outbreak size.^11^

While conceptually sound, it is unclear if such a serological testing-based strategy is viable for college campuses. First, the amount of protection provided by a given level of antibodies, which the test measures, is unknown.^29^ Second, it is uncertain for how long immunity might last.^30,31^ There is evidence that immunity against SARS-CoV-2 might wane quickly. A study found that 40% (12/30) of asymptomatic individuals and 12.9% (4/31) of symptomatic individuals became IgG-negative 8 weeks after discharge from hospitals.^32^ Third, false positives are a concern.^31,33^ If an individual is placed into an “immune shield” position despite not actually being immune, this could lead to an increase in transmission.^28^ Fourth, preliminary results of seroprevalence studies suggest that immune individuals will be in short supply. A meta-analysis of multiple seroprevalence studies using Bayesian hierarchical models,^34^ found that seroprevalence is low among the populations investigated, with 2-6% in Los Angeles County, California,^35^ 0-2% in Santa Clara County, California,^36^ 1.6-2.6% in San Miguel County, Colorado, and 15-41% in Chelsea, Massachusetts. Cautious interpretation of studies where participants were recruited by means of social media advertisement as a convenience sample,^36^ is needed given potential selection bias; accounting for the uncertainty in the sensitivity and specificity of the tests is also recommended.^37,38^ Concerns remain whether studies in certain COVID-19 hotspots are representative of the general population nationwide. It is likely that, in August 2020, universities can expect <10% of returning students being potentially immune while the majority will remain susceptible. If 5% of a campus community were to be seropositive, this would mean 1,000 individuals on a campus of 20,000. If these seropositive individuals could be deployed to have 10 times as many contacts as other groups, it would lead to a reduction of ~31% in transmission, not enough to get the reproduction number below 1.^11^

Furthermore, the logistical challenges are obvious. When semester begins, everyone needs to get tested rapidly. Then, seropositive individuals will be trained to fill positions with high contact rates (eg, campus bus drivers, food service workers, residential facility managers). Both matching positions to talents and on-the-job training take time. Cost is another concern. Medicare pays $42-45 per SARS-CoV-2 serological test.^20^ This translates into a price tag of $840,000-$900,000 for a 20,000-strong campus. Ethical issues have also been raised.^39^ If student employment opportunities are limited, and they are open to immune people only, would students deliberately get themselves infected as in “COVID-19 parties”^40^ so that they can take up those jobs? Conversely, there are media reports that some recovered individuals have been stigmatized.^31^ It is also known that, currently, vulnerable minorities are more likely to be infected.^41^ Would it be equitable to ask those individuals who were infected because they were unable to shelter-at-home to perform extra duties as “immune shields”? Thus, with all these logistical, financial, and ethical concerns, while the serological testing and immune shield strategy might be feasible in subpopulations such as health-care workers, it appears not to be a promising control strategy for college campuses.

## CONCLUSIONS

We discussed some practical considerations for 2 potential testing-based control strategies as applied to college campus reopening: RT-PCR testing followed by case-patient isolation and quarantine of close contacts; and serological testing followed by “immune shields.” Given the logistic and ethical issues, neither approach appears to be viable as a main strategy. We do believe they could be used as supplementary strategies or applied to specific groups (eg, individuals with high contact rates). Unless the issues we discussed above can be resolved in such a way that using mass testing as main control strategies becomes viable, the main nonpharmaceutical interventions will remain methods based on social distancing and personal protective behaviors until vaccines become available. Online or hybrid instruction this fall will significantly reduce the risk of on-campus outbreaks.^42^ For those planning for in-person instruction,^8^ social distancing measures, including capping class size at a small number,^43^ and the adoption of personal protective behaviors, including universal facemask wearing in public space,^44^ will be required to reduce—but not eliminate—on-campus transmission.^24^ Given the ongoing debate in some university systems whether to make facemask-wearing in public mandatory,^45^ and the challenges of enforcing social distancing among students outside the classroom setting, there is a sizeable risk of on-campus COVID-19 outbreaks when in-person instruction resumes on college campuses this fall.^24^

## References

[1] Moore KA , Lipsitch M , Barry JM , Osterholm MT. COVID-19: the CIDRAP viewpoint. Part 1: The future of the COVID-19 pandemic: lessons learned from pandemic influenza. 2020. https://www.cidrap.umn.edu/sites/default/files/public/downloads/cidrap-covid19-viewpoint-part1_0.pdf. Accessed May 17, 2020.

[2] Centers for Disease Control and Prevention. COVID-19 Forecasts. 2020. https://www.cdc.gov/coronavirus/2019-ncov/covid-data/forecasting-us.html. Accessed May 14, 2020.

[3] Fung IC , Antia R , Handel A. How to minimize the attack rate during multiple influenza outbreaks in a heterogeneous population. PLoS One. 2012;7(6):e36573. 22701558 10.1371/journal.pone.0036573 PMC3372524

[4] Handel A , Longini IM Jr , Antia R. What is the best control strategy for multiple infectious disease outbreaks? Proc Biol Sci. 2007;274(1611):833-837. 17251095 10.1098/rspb.2006.0015 PMC2093965

[5] Daniels ME Jr. A message from President Daniels regarding fall semester (April 21, 2020). *Purdue University.* 2020. https://www.purdue.edu/president/messages/campus-community/2020/2004-fall-message.php. Accessed May 14, 2020.

[6] Struck K. Students sue Liberty University over COVID-19 response (April 14, 2020). *Voice of America.* 2020. https://www.voanews.com/science-health/coronavirus-outbreak/students-sue-liberty-university-over-covid-19-response. Accessed May 14, 2020.

[7] Fain P. The evolving fall picture (April 29, 2020). *Inside Higher Ed.* 2020. https://www.insidehighered.com/news/2020/04/29/growing-number-colleges-announce-intent-reopen-fall. Accessed May 5, 2020.

[8] Chronicle Staff . Here’s a list of colleges’ plans for reopening in the fall (Last updated on June 22, 2020). *The Chronical of Higher Education.* 2020. https://www.chronicle.com/article/Here-s-a-List-of-Colleges-/248626. Accessed June 23, 2020.

[9] American College Health Association . ACHA guidelines: considerations for reopening institutions of higher education in the COVID-19 era (May 7, 2020). 2020. https://www.acha.org/documents/resources/guidelines/ACHA_Considerations_for_Reopening_IHEs_in_the_COVID-19_Era_May2020.pdf. Accessed May 20, 2020.

[10] Centers for Disease Control and Prevention. Considerations for institutes of higher education (Updated May 21, 2020). 2020. https://www.cdc.gov/coronavirus/2019-ncov/community/colleges-universities/considerations.html. Accessed May 22, 2020.

[11] Weitz JS , Beckett SJ , Coenen AR , et al. Modeling shield immunity to reduce COVID-19 epidemic spread. Nat Med. 2020;26(6):849-854. 32382154 10.1038/s41591-020-0895-3 PMC8272982

[12] University of California San Diego. Return to learn program. 2020. https://coronavirus.ucsd.edu/return-to-learn/index.html. Accessed May 25, 2020.

[13] He X , Lau EHY , Wu P , et al. Temporal dynamics in viral shedding and transmissibility of COVID-19. Nat Med. 2020;26(5):672-675. 32296168 10.1038/s41591-020-0869-5

[14] Wu JT , Leung K , Leung GM. Nowcasting and forecasting the potential domestic and international spread of the 2019-nCoV outbreak originating in Wuhan, China: a modelling study. Lancet. 2020;395(10225):689-697. 32014114 10.1016/S0140-6736(20)30260-9 PMC7159271

[15] Lauer SA , Grantz KH , Bi Q , et al. The incubation period of coronavirus disease 2019 (COVID-19) from publicly reported confirmed cases: estimation and application. Ann Intern Med. 2020;172(9):577-582. 32150748 10.7326/M20-0504 PMC7081172

[16] Centers for Disease Control and Prevention. COVID-19 pandemic planning scenarios. 2020 https://www.cdc.gov/coronavirus/2019-ncov/hcp/planning-scenarios.html. Accessed May 27, 2020.

[17] Liu Y , Centre for Mathematical Modelling of Infectious Diseases nCoV Working Group, Funk S, Flasche S. The contribution of pre-symptomatic infection to the transmission dynamics of COVID-2019 [version 1; peer review: 1 approved]. Wellcome Open Res. 2020;5:58. 32685697 10.12688/wellcomeopenres.15788.1 PMC7324944

[18] Centers for Disease Control and Prevention. Quarantine and isolation (May 6 version). 2020. https://www.cdc.gov/coronavirus/2019-ncov/if-you-are-sick/quarantine-isolation.html. Accessed May 15, 2020.

[19] Nadworny E. For In-person college, coronavirus testing will be key. But is that feasible? *National Public Radio.* 2020. https://www.npr.org/2020/05/22/858601308/for-in-person-college-coronavirus-testing-will-be-key-but-is-that-feasible. Accessed May 22, 2020.

[20] Centers for Medicare and Medicaid Services. Medicare Administrative Contractor (MAC) COVID-19 Test Pricing (May 19, 2020). 2020. https://www.cms.gov/files/document/mac-covid-19-test-pricing.pdf. Accessed May 19, 2020.

[21] Wang Y , Wang Y , Chen Y , et al. Unique epidemiological and clinical features of the emerging 2019 novel coronavirus pneumonia (COVID-19) implicate special control measures. J Med Virol. 2020;92(6):568-576. doi:10.1002/jmv.25748 32134116 10.1002/jmv.25748 PMC7228347

[22] Ai T , Yang Z , Hou H , et al. Correlation of chest CT and RT-PCR testing in coronavirus disease 2019 (COVID-19) in China: a report of 1014 cases. Radiology. 2020;296(2):E32-E40. 32101510 10.1148/radiol.2020200642 PMC7233399

[23] Stein R. Study raises questions about false negatives from quick COVID-19 test (April 21, 2020). *National Public Radio.* 2020. https://www.npr.org/sections/health-shots/2020/04/21/838794281/study-raises-questions-about-false-negatives-from-quick-covid-19-test. Accessed May 19, 2020.

[24] Gressman PT , Peck JR. Simulating COVID-19 in a university environment. *arXiv.* 2020:2006.03175. https://arxiv.org/abs/2006.03175. Accessed July 29, 2020. 10.1016/j.mbs.2020.108436 PMC7398032 32758501

[25] Gao Z , Xu Y , Sun C , et al. A systematic review of asymptomatic infections with COVID-19. J Microbiol Immunol Infect. 2020. doi: 10.1016/j.jmii.2020.05.001 PMC7227597 32425996

[26] Hall MA , Studdert DM. Privileges and immunity certification during the COVID-19 pandemic. JAMA. 2020;323(22):2243-2244. 10.1001/jama.2020.7712 32374358

[27] Persad G , Emanuel EJ. The ethics of COVID-19 immunity-based licenses (“Immunity Passports”). JAMA. 2020;323(22):2241-2242. 10.1001/jama.2020.8102 32374357

[28] Kraay ANM , Nelson K , Zhao C , et al. Modeling serological testing to inform relaxation of social distancing for COVID-19 control. *medRxiv.* 2020.Version 2. https://www.medrxiv.org/content/10.1101/2020.04.24.20078576v2. Accessed July 29, 2020. 10.1038/s41467-021-26774-y PMC8642547 34862373

[29] Clapham H , Hay J , Routledge I , et al. Seroepidemiologic study designs for determining SARS-COV-2 transmission and immunity. Emerg Infect Dis. 2020;26(9). doi:10.3201/eid2609.201840 PMC7454079 32544053

[30] Harris R. Will antibodies after COVID-19 illness prevent reinfection? (May 7, 2020). *National Public Radio.* 2020. https://www.npr.org/sections/health-shots/2020/05/07/852360101/will-antibodies-after-covid-19-illness-prevent-reinfection. Accessed May 14, 2020.

[31] Karimi F. Coronavirus ‘immunity passports’ are a terrible idea that could backfire, experts warn (May 22, 2020). *CNN.* 2020; https://www.cnn.com/2020/05/22/health/immunity-passport-coronavirus/index.html. Accessed May 22, 2020.

[32] Long Q-X , Tang X-J , Shi Q-L , et al. Clinical and immunological assessment of asymptomatic SARS-CoV-2 infections. Nat Med. 2020. doi: 10.1038/s41591-020-0965-6. 32555424

[33] Cairns E. Roche takes on Abbott in Covid-19 antibody testing (May 4, 2020). 2020. https://www.evaluate.com/vantage/articles/news/policy-and-regulation/roche-takes-abbott-covid-19-antibody-testing. Accessed May 25, 2020.

[34] Levesque J , Maybury DW. A note on COVID-19 seroprevalence studies: a meta-analysis using hierarchical modelling (Version 1). MedRxiv. 2020 10.1101/2020.05.03.20089201. Accessed June 24, 2020.

[35] Sood N , Simon P , Ebner P , et al. Seroprevalence of SARS-CoV-2-specific antibodies among adults in Los Angeles County, California, on April 10-11, 2020. JAMA. 2020;323(23):2425-2427. 10.1001/jama.2020.8279 PMC7235907 32421144

[36] Bendavid E , Mulaney B , Sood N , et al. COVID-19 antibody seroprevalence in Santa Clara County, California. *medRxiv*. 2020. Version 2. doi:10.1101/2020.04.14.20062463 PMC7928865 33615345

[37] Gelman A , Carpenter B. Bayesian analysis of tests with unknown specificity and sensitivity (Version 2). medRxiv. 2020. doi: 10.1101/2020.05.22.20108944 PMC10016948 37252679

[38] Bennett ST , Steyvers M. Estimating COVID-19 antibody seroprevalence in Santa Clara County, California. A re-analysis of Bendavid et al. medRxiv. 2020. doi: 10.1101/2020.04.24.20078824 PMC7928865 33615345

[39] Voo TC , Clapham H , Tam CC. Ethical implementation of immunity passports during the COVID-19 pandemic. J Infect Dis. 2020;222(5):715-718. https://academic.oup.com/jid/article/doi/10.1093/infdis/jiaa352/5862418. 32582943 10.1093/infdis/jiaa352 PMC7337820

[40] Baker M. ‘Covid-19 Parties’ probably didn’t involve intentional spread (May 6, 2020). *New York Times.* 2020. https://www.nytimes.com/2020/05/06/us/coronavirus-covid-parties.html. Accessed May 14, 2020.

[41] Dyer O. Covid-19: black people and other minorities are hardest hit in US. BMJ. 2020;369:m1483. 32291262 10.1136/bmj.m1483

[42] Huang PH , Austin DS. Unsafe at any campus: don’t let colleges become the next cruise ships, nursing homes, and meat packing plants. Indiana Law J Suppl. 2020. doi: 10.2139/ssrn.3612467

[43] Weeden KA , Cornwell B. The small world network of college classes: implications for epidemic spread on a university campus. Sociol Sci. 2020;7:222-241.

[44] Dhillon RS , Karan A , Beier D , et al. A plan to safely reopen the U.S. despite inadequate testing. *Harvard Business Review.* 2020 https://hbr.org/2020/05/a-plan-to-safely-reopen-the-u-s-despite-inadequate-testing. Accessed May 1, 2020.

[45] Shearer L. Peition asks UGA, other state colleges to require masks in fall (June 22, 2020). *Athens Banner-Herald.* 2020. https://www.onlineathens.com/news/20200622/petition-asks-uga-other-state-colleges-to-require-masks-in-fall. Accessed June 23, 2020.

